# Improved risk estimation of locoregional recurrence, secondary contralateral tumors and distant metastases in early breast cancer: the INFLUENCE 2.0 model

**DOI:** 10.1007/s10549-021-06335-z

**Published:** 2021-08-02

**Authors:** Vinzenz Völkel, Tom A. Hueting, Teresa Draeger, Marissa C. van Maaren, Linda de Munck, Luc J. A. Strobbe, Gabe S. Sonke, Marjanka K. Schmidt, Marjan van Hezewijk, Catharina G. M. Groothuis-Oudshoorn, Sabine Siesling

**Affiliations:** 1 Tumor Center Regensburg/University of Regensburg, Institute for Quality Control and Health Services Research, Regensburg, Germany; 2 Evidencio, medical Decision Support, Haaksbergen, The Netherlands; 3 Department of Health Technology and Services Research, Technical Medical Centre, University of Twente, POBox 217, Enschede, 7500 AE The Netherlands; 4 Department of Research and Development, Netherlands Comprehensive Cancer Organisation (IKNL), POBox 19079, Utrecht, 3501 DB The Netherlands; 5 Department of Surgical Oncology, Canisius Wilhelmina Hospital, Nijmegen, The Netherlands; 6 Department of Medical Oncology, Netherlands Cancer Institute, Amsterdam, The Netherlands; 7 Division of Molecular Pathology, Netherlands Cancer Institute—Antoni van Leeuwenhoek, Amsterdam, The Netherlands; 8 Institution for Radiation Oncology, Arnhem, The Netherlands

**Keywords:** Mamma carcinoma, Risk prediction, Follow-up, Recurrence, Contralateral breast cancer, Metachronous metastasis

## Abstract

**Purpose:**

To extend the functionality of the existing INFLUENCE nomogram for locoregional recurrence (LRR) of breast cancer toward the prediction of secondary primary tumors (SP) and distant metastases (DM) using updated follow-up data and the best suitable statistical approaches.

**Methods:**

Data on women diagnosed with non-metastatic invasive breast cancer were derived from the Netherlands Cancer Registry (*n* = 13,494). To provide flexible time-dependent individual risk predictions for LRR, SP, and DM, three statistical approaches were assessed; a Cox proportional hazard approach (COX), a parametric spline approach (PAR), and a random survival forest (RSF). These approaches were evaluated on their discrimination using the Area Under the Curve (AUC) statistic and on calibration using the Integrated Calibration Index (ICI). To correct for optimism, the performance measures were assessed by drawing 200 bootstrap samples.

**Results:**

Age, tumor grade, pT, pN, multifocality, type of surgery, hormonal receptor status, HER2-status, and adjuvant therapy were included as predictors. While all three approaches showed adequate calibration, the RSF approach offers the best optimism-corrected 5-year AUC for LRR (0.75, 95%CI: 0.74–0.76) and SP (0.67, 95%CI: 0.65–0.68). For the prediction of DM, all three approaches showed equivalent discrimination (5-year AUC: 0.77–0.78), while COX seems to have an advantage concerning calibration (ICI < 0.01). Finally, an online calculator of INFLUENCE 2.0 was created.

**Conclusions:**

INFLUENCE 2.0 is a flexible model to predict time-dependent individual risks of LRR, SP and DM at a 5-year scale; it can support clinical decision-making regarding personalized follow-up strategies for curatively treated non-metastatic breast cancer patients.

**Supplementary Information:**

The online version contains supplementary material available at 10.1007/s10549-021-06335-z.

## Introduction

In the Netherlands, more than 14,000 women per year are diagnosed with invasive breast cancer [1], rendering it the most frequently diagnosed malignancy among women [2]. Early detection and advanced treatment strategies have led to improved survival during the last decade [3–5]. The current average 5-year survival rate of women diagnosed with breast cancer (all stages) is 88% in the Netherlands [6]. The Dutch breast cancer guideline recommends annual mammograms and physical examinations during the first five years following curative treatment, unless bilateral mastectomy was performed [7]. This follow-up program is uniform for all patients and does not take individual risk profiles into account. To avoid unnecessary follow-up visits and examinations possibly inflicting psychological harm [8–10] and causing additional societal costs, the creation of personalized follow-up patterns based on individual risk estimations would be reasonable.

In 2015, Witteveen et al. [11] developed the “INFLUENCE nomogram”, which estimates an individual breast cancer patient’s five-year recurrence risk as well as conditional annual risks of developing a local or regional recurrence based on different patient, tumor and treatment characteristics. Thus, it can be used to support clinical decision-making; nevertheless, it neglects some relevant factors: Breast cancer follow-up aims not only at the detection of locoregional recurrences (LRR) but also of secondary primary contralateral breast tumors (SP) [7]. Additionally, an estimate of the risk for developing metachronous distant metastasis (DM) is relevant for understanding a patient’s prognosis and might influence decision-making regarding an optimal follow-up strategy. Besides, the HER2-status is not among the predictors of the current INFLUENCE nomogram although it has a considerable influence on therapy decisions [12, 13]. From a statistical point of view, the INFLUENCE nomogram is based on five logistic regression models yielding risk estimations for the subsequent year at five arbitrary fixed time points. Other statistical approaches may contribute to improve its performance. For routine implementation in clinical practice, more detailed risk estimations for periods with flexible length are required. Thus, the patients’ (changing) need for customized information could be better served and it would be possible to tailor follow-up schemes exactly to the development of individual risk profiles over time.

Aiming to incorporate all these factors, it was our aim to update the existing INFLUENCE nomogram toward an advanced INFLUENCE 2.0 model based on a large Dutch nationwide cohort. This new model is supposed to predict flexible time-dependent individual risks of LRR, SP, and DM in curatively treated non-metastatic breast cancer patients. To make the development of INFLUENCE 2.0 transparent, this paper describes the selection process among three candidates for the optimal statistical approach and describes the performance of the final model, which will be made available online in a user friendly calculator.

## Methods

### Study population and variables

Data for the development of the INFLUENCE 2.0 model were derived from the Netherlands Cancer Registry (NCR), a nationwide database collecting records of all newly diagnosed malignant tumors in the country hosted by the Netherlands Comprehensive Cancer Organisation (IKNL) since 1989. After notification through the nationwide pathology archive (PALGA), information on each patient is collected by specially trained registration clerks directly from patient files. The data include patient demographics, tumor-, and treatment characteristics. Vital status and date of death are regularly retrieved through linkage with the national municipality registry. Using the NCR database, we selected all women with non-metastatic (pT1-3, any pN) primary invasive adenocarcinoma of the breast, diagnosed in 2007, 2008 or the first quarter of 2012. For this cohort, active follow-up for the first five years following successful removal of the primary tumor was conducted and information on recurrences occurring within five years from diagnosis was collected. Patients were excluded in case of positive resection margins of the primary tumor, if a neoadjuvant therapy was conducted, or if surgery took place later than 180 days after diagnosis. Missing data were assumed to be missing at random. Therefore, only patients without missing data concerning potential predictor variables were included.

The INFLUENCE 2.0 model aims to estimate individual time-dependent risks for three types of events, defined according to consensus-based definitions [14]:

- Locoregional recurrence, LRR, defined as reappearance of the tumor in the ipsilateral breast, chest wall or regional lymph nodes
- Second Primary breast cancer, SP, defined as secondary primary tumor of the contralateral breast
- Distant metastasis, DM, defined as pathologically or radiologically confirmed reappearance of tumor tissue at any location in the body

An individual is regarded to be at risk for any of these events starting the day following radical surgical removal of the primary tumor. In case of multiple events, only the first event was considered.

The following variables were selected as predictors for the named events based on previous studies and clinical expertise: age, pT-stage, pN-stage, multifocality, grading, hormone receptor status (estrogen receptor (ER)- and progesterone receptor (PR)-status), antihormonal therapy, human epidermal growth factor receptor 2 (HER2-status), type of surgery, adjuvant chemotherapy, adjuvant radiation therapy and antibody therapy. Since hormone receptor status and antihormonal therapy are highly dependent on each other (e.g., patients with negative ER-status do obviously not receive antihormonal therapy), the predictors were merged. A similar linkage exists between HER2-status and antibody therapy.

### Model development

The INFLUENCE 2.0 model was designed to enable its users to choose a prediction period of variable length within five years after successful primary surgery. To optimize model performance, three statistical approaches were tested to find the best-performing model algorithm: A Cox proportional hazards approach (COX), a parametric spline approach (PAR), and a random survival forest (RSF):

- The Cox proportional hazards approach [15] is regarded a semiparametric model since it does not assume any particular baseline survival distribution. However, it takes for granted that the predictors have a fixed effect on the underlying hazard function.
- If a changing effect of one or more predictor variables over time is assumed, the parametric spline approach might be a better choice. Basically, it consists of several piecewise defined spline functions which are joined in so-called “knots”. In every piece, the influence of a predictor on the hazard function can be different.
- The Random Survival Forest [16, 17] is an extension of the classical Random Forest concept for binary outcomes [18] to analyze right censored time-to-event data. A forest of survival trees is grown using a log-rank splitting rule to select the optimal predictor variables. Survival estimates are constructed with a Kaplan–Meier estimator [19] within each terminal node, at each time.

### Model performance

The three potential statistical approaches were validated and compared on their predictive ability using performance measures for calibration and discrimination [20].

Calibration concerns the congruence between observed and predicted events. To provide quantified summary measures of model calibration, the Integrated Calibration Index (ICI, weighted average), E50 (median) and E90 (90th percentile) were calculated at *t* = 1, 2, 3, 4, and 5 years. These measures denote the absolute difference between observed and predicted probabilities [21].

Discrimination was quantified using the area under the receiver operating characteristic curve (AUC). The AUC reflects the probability of a random sample of individuals with an event having a higher predicted risk than a random sample of individuals without an event. An AUC of 1.0 indicates perfect discrimination, whereas 0.5 is equal to chance. The AUC was measured based on a quarterly time frame over the whole five-year prediction period to assess the three approaches’ difference in AUC over time using a cumulative/dynamic approach as described by Kamarudin et al. [22].

The performance measures were obtained as apparent and adjusted values. The apparent results reflect the performance of the tested approaches in the same data used to train them. Additionally, adjusted performance measures were estimated in 200 bootstrap samples. They reflect the performance of an approach trained in a bootstrap sample applied on the entire dataset. The difference between apparent and adjusted performance denotes the level of optimism. A low level of optimism indicates a more robust performance. The adjusted results represent the optimism-corrected performance and were used to decide upon the optimal statistical approach for the final model [23].

Ultimately, INFLUENCE 2.0 is meant to support the tailoring of optimal individual follow-up strategies aiming at the detection of LRR and SP as potentially curable events. Therefore, discrimination was selected as key measure in the comparison of the three tested statistical approaches predicting these events. In contrast to this, knowing the risk of DM can only serve an informative purpose; predicting DM means predicting the risk of a palliative situation in which classical follow-up would not make sense, anymore. Consequently, calibration was considered the central indicator in selecting the most appropriate statistical approach to predict this event.

### Software and online model

For the analyses, R version 3.6.3 (R Foundation for Statistical Computing, Vienna, Austria; http://www.R-project.org/) was used. To develop the RSF, COX and PAR algorithms, the packages “randomForestSRC [24], “survival” [25, 26], and “rstpm2” [27, 28] were used. For the performance analyses, we employed the packages “timeROC” [29], and “boot” [30]. Based on the best-performing statistical approach, an online calculator of the INFLUENCE 2.0 model was developed and made available on www.evidencio.org, an online platform for medical prediction models.

## Results

### Descriptive statistics

In total, 17,014 patients with an invasive adenocarcinoma of the breast diagnosed in 2007, 2008, or the first quarter of 2012 were identified from the NCR. Of those, 13,494 met all eligibility criteria. Supplementary figure S1 gives a detailed overview of the exclusion process.

All relevant characteristics of the patient cohort are shown in Table 1. The majority of the patients (98%) had a pT1 or pT2 tumor, no lymph node involvement (65%), low tumor grade (70% grade 1 or 2), and a unifocal tumor (85%). In about 60% of the cases, breast-conserving surgery was performed. Adjuvant radiation therapy and adjuvant chemotherapy were administered in 67% and 38% of the patients, respectively. Over 80% of the patients were ER and/or PR positive and about 40% of them received antihormonal therapy. Of all patients, less than 15% were Her2-positive, of whom 60% received antibody-treatment. Within five years, 385 (2.8%), 411 (3.0%), and 848 (6.3%) patients developed a LRR, SP, or DM, respectively, as their first event. A total of 11,839 (87.7%) remained free of recurrence.

**Table 1:** Patient characteristics

Variable	*N* (%) total = 13,494
Inclusion year	
Inclusion year = 2007	5508 (41.1%)
Inclusion year = 2008	5621 (41.7%)
Inclusion year = 2012	2365 (17.5%)
Age-group	
50–59	3091 (22.9%)
60–69	3635 (26.9%)
70–79	3531 (26.2%)
≥ 80	3237 (24.0%)
Grading	
1	3409 (25.3%)
2	6047 (44.8%)
3	4038 (29.9%)
pT	
pT1	8692 (64.4%)
pT2	4514 (33.5%)
pT3	288 (2.1%)
pN	
pN0	8782 (65.1%)
pN1	3493 (25.9%)
pN2	790 (5.9%)
pN3	429 (3.2%)
Multifocality	
No	11,425 (84.7%)
Yes	2069 (15.3%)
Surgery	
Breast-conserving surgery	7942 (58.9%)
Mastectomy	5552 (41.1%)
Chemotherapy	
No	8366 (62%)
Yes	5128 (38%)
Radiotherapy	
No	4403 (32.6%)
Yes	9091 (67.4%)
Hormonal therapy	
HR + & no therapy	6560 (48.6%)
HR + & therapy	4881 (36.2%)
HR−	2053 (15.2%)
Targeted therapy	
HER2 + & no therapy	678 (5.0%)
HER2 + & therapy	1015 (7.5%)
HER2−	11,801 (87.5%)
First event	
LRR	385 (2.8%)
SP	411 (3.0%)
DM	848 (6.3%)
None	11,839 (87.7%)

*N* number of patients, *pT* pathological tumor stage, p*N* pathological nodal stage, *LRR* Locoregional Recurrence, *SP* Secondary Primary, *DM* Distant metastasis

### Internal validation and comparison of modeling approach

**Fig. 1:**
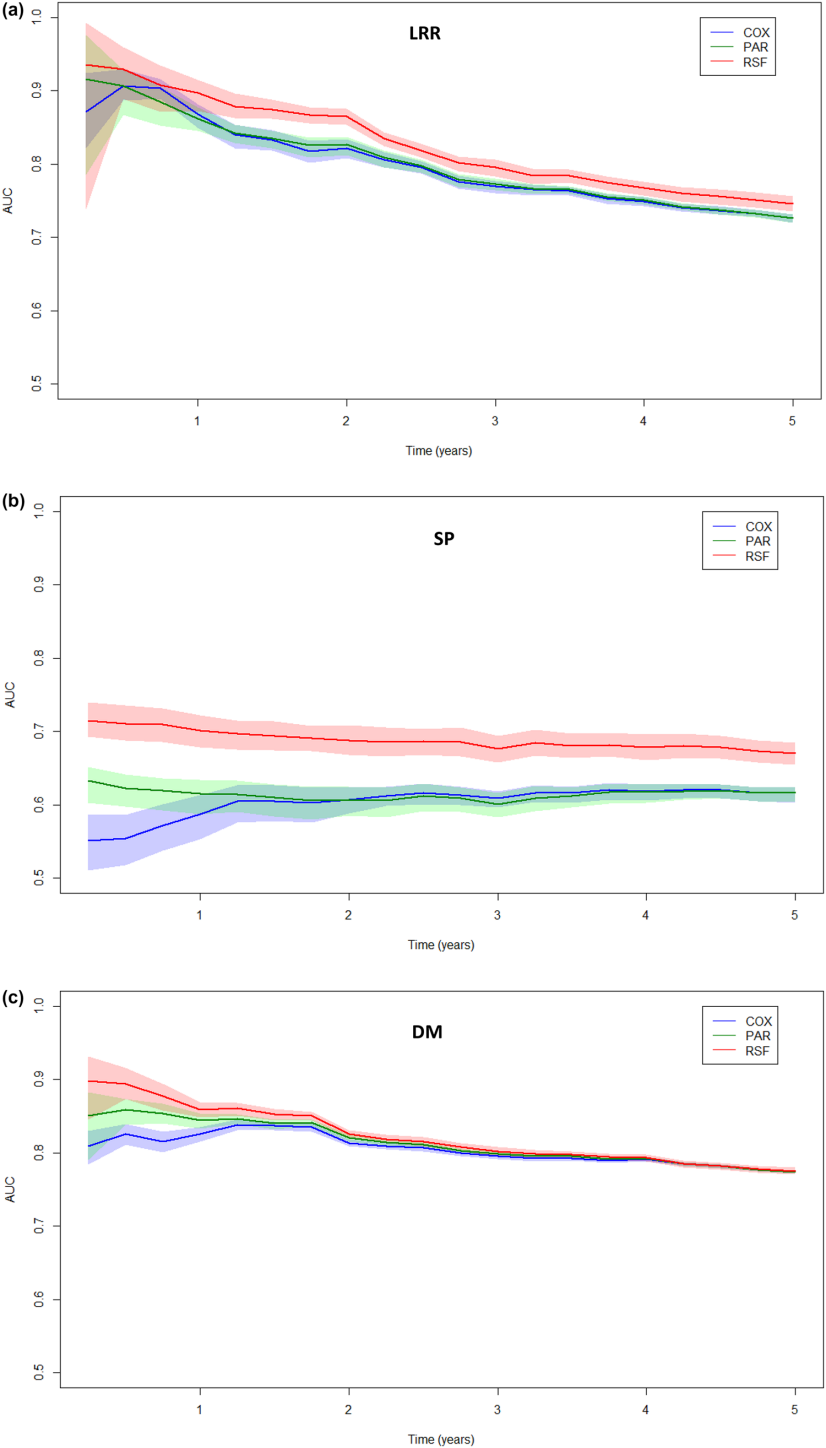
**a** Area under the Receiver operating characteristic curve (AUC) per quarter year for the outcome locoregional recurrence (LRR). The lines are displayed with 95% Confidence Intervals. **b** Area under the Receiver operating characteristic curve (AUC) per quarter year for the outcome secondary primary (SP). The lines are displayed with 95% Confidence Intervals. **c** Area under the Receiver operating characteristic curve (AUC) per quarter year for the outcome distant metastasis (DM). The lines are displayed with 95% Confidence Intervals. *Cox* Cox proportional hazard model, *PAR* Parametric Spline Model, and *RSF* Random Survival Forest

For the prediction of LRR, the optimism-corrected discrimination is displayed graphically in Fig. 1a. The RSF approach shows significantly higher AUC values after the first year until the end of year five compared to the COX and PAR approaches. The AUCs of the COX, PAR, and RSF models at year five were 0.73 (95%CI: 0.72–0.73), 0.73 (95%CI: 0.72–0.73), and 0.75 (95%CI: 0.74–0.76), respectively. On average, the optimism in the AUCs was higher for the RSF approach (optimism = 0.04) than for the PAR approach (optimism = 0.02) and the Cox approach (optimism = 0.01). Calibration is displayed in Table 2; it shows that all three modeling approaches show adequate calibration at all tested time points, reflected by an ICI, E50, and E90 below 0.01. Based on these outcomes, the RSF was selected for the final INFLUENCE 2.0 model as optimal approach to predict the risk for LRR.

**Table 2:** Calibration results

Outcome	Time (years)	COX			PAR			RSF		
		ICI	E50	E90	ICI	E50	E90	ICI	E50	E90
Locoregional	1	0.0005	0.0003	0.0007	0.0019	0.0014	0.0016	**0.0009**	**0.0006**	**0.0015**
	2	0.0011	0.0008	0.0016	0.0030	0.0021	0.0025	**0.0023**	**0.0018**	**0.0033**
	3	0.0017	0.0013	0.0027	0.0028	0.0022	0.0032	**0.0044**	**0.0023**	**0.0039**
	4	0.0023	0.0018	0.0037	0.0023	0.0019	0.0036	**0.0064**	**0.0035**	**0.0055**
	5	0.0027	0.0022	0.0044	0.0028	0.0021	0.0042	**0.0094**	**0.0057**	**0.0096**
Second primary	1	0.0010	0.0009	0.0017	0.0030	0.0024	0.0060	**0.0010**	**0.0008**	**0.0017**
	2	0.0013	0.0011	0.0023	0.0020	0.0016	0.0036	**0.0018**	**0.0014**	**0.0032**
	3	0.0016	0.0015	0.003	0.0022	0.0021	0.0033	**0.0037**	**0.0028**	**0.0063**
	4	0.0021	0.0018	0.0038	0.0021	0.0019	0.0036	**0.0049**	**0.004**	**0.0088**
	5	0.0025	0.0022	0.0044	0.0026	0.0022	0.0047	**0.0073**	**0.0059**	**0.0135**
Distant metastasis	1	**0.0007**	**0.0004**	**0.0012**	0.0030	0.0024	0.0034	0.0014	0.0008	0.0017
	2	**0.0017**	**0.0009**	**0.0024**	0.0055	0.0035	0.0062	0.0060	0.0036	0.0058
	3	**0.0026**	**0.0015**	**0.0038**	0.0054	0.0035	0.0075	0.0119	0.0074	0.0123
	4	**0.0032**	**0.0020**	**0.0045**	0.0043	0.0028	0.0075	0.0166	0.0106	0.0201
	5	**0.0037**	**0.0024**	**0.0054**	0.0033	0.0024	0.0054	0.0216	0.0143	0.0299

The displayed results represent the optimism-corrected values. The ICI is the integrated calibration index, E50 is the median absolute difference between observed and expected, and E90 is the 90th percentile of the absolute difference. Bold results display the results for the models that were selected as the best-performing model. For LRR, and SP, the decision was primarily based on the discrimination

For the prediction of SP, the RSF approach shows superior performance concerning discrimination compared to the other approaches at all time points (Fig. 1b). The optimism-corrected AUCs at year five for the COX, PAR, and RSF approaches were 0.62 (95%CI: 0.60–0.62), 0.62 (95%CI: 0.60–0.62), 0.67 (95%CI: 0.65–0.68), respectively. On average, the optimism in the AUCs for the RSF approach was higher (optimism = 0.08) than for the PAR approach (optimism = 0.03) and the Cox approach (optimism = 0.02). Calibration is displayed in Table 2 and shows that all three modeling approaches show adequate calibration at all tested time points, reflected by an ICI, E50, and E90 below 0.01, with an exception for the RSF approach at year 5 (E90 = 0.0135). Finally, the RSF was selected as best-performing approach to predict the risk for SP.

For the prediction of DM, calibration is displayed in Table 2; it shows that the COX approach proofed to be best calibrated. Generally, all three statistical approaches showed mostly adequate calibration at each of the tested annual time points, reflected by an ICI below 0.01. However, the RSF approach seems to be associated with a lower level of accuracy at some time points. For the years 3, 4, and 5 its ICI is 0.012, 0.017, and 0.022, respectively. The performance of the approaches concerning discrimination is displayed in Fig. 1c. With exception of the first year, all three modeling approaches showed similar performance on discrimination. The optimism-adjusted AUCs at year five for the COX, PAR, and RSF approaches were 0.77 (95%CI: 0.77–0.78), 0.77 (95%CI: 0.77–0.78), and 0.78 (95%CI: 0.77–0.78), respectively. On average, the optimism in the AUCs for the RSF approach was higher (optimism = 0.02) than for the PAR approach (optimism = 0.01) and the Cox approach (optimism = 0.004). Based on these results, the COX approach was selected for the final INFLUENCE 2.0 model to predict the risk for DM. Table 3 gives an overview of the underlying coefficients.

**Table 3:** Coefficients of the Cox regression model selected to predict DM

Variable	Option	Hazard ratio	95% CI
Age	< 60	Reference	
	60–70	0.916	0.7530–1.1133
	70–80	0.841	0.6775–1.0443
	≥ 80	1.240	0.9693–1.5857
Grade	I	Reference	
	II	2.359	1.7941–3.1004
	III	4.081	3.0610–5.4404
Tumor stage	pT1	Reference	
	pT2	2.280	1.9338–2.688
	pT3	2.499	1.7857–3.4976
Nodal stage	pN0	Reference	
	pN1	1.879	1.5714–2.2469
	pN2	4.109	3.2221–5.2395
	pN3	7.503	5.8160–9.6785
Multifocality	No	Reference	
	Yes	1.242	1.0426–1.4783
Surgery	Breast-conserving surgery	Reference	
	Mastectomy	0.915	0.7405–1.1302
Chemotherapy	No	Reference	
	Yes	0.676	0.5436–0.8413
Radiotherapy	No	Reference	
	Yes	0.913	0.7332–1.1371
Hormone receptor status & treatment	Negative	Reference	
	Positive with treatment	0.506	0.4258–0.6014
	Positive without treatment	0.751	0.5868–0.9603
HER2-status & treatment	Negative	Reference	
	Positive with treatment	0.704	0.5301–0.9360
	Positive without treatment	1.188	0.9634–1.4642
	Time		
Baseline hazard	Year 1	0.002417	
	Year 2	0.007263	
	Year 3	0.012676	
	Year 4	0.017466	
	Year 5	0.021168	

### Online calculator

The final INFLUENCE 2.0 model returns risk predictions for LRR, SP, and DM based on the selected statistical approaches; an easy-to-use online risk calculator is available via: https://www.evidencio.com/models/show/2238. The online calculator estimates the risks and the 95% confidence intervals based on the 200 bootstrapped models.

## Discussion

In this study, we developed a model predicting the risks for LRR, SP, and DM within 5 years after primary surgery for patients with curatively treated non-metastatic breast cancer. For this purpose, three different statistical approaches were compared concerning discrimination and calibration. Based on discrimination, the RSF approach showed superior performance in the prediction of LRR and SP as compared to COX and PAR. However, the COX approach showed a higher level of agreement between the predicted and observed risks, which was decisive for the selection of the best-performing approach for the prediction of DM, as the discriminatory performance concerning this event was similar between the modeling approaches.

### Comparison to the original INFLUENCE nomogram and other related prediction models

Compared to the original INFLUENCE nomogram, the INFLUENCE 2.0 model comes with a variety of updates leading to improved flexibility and a broader application range regarding predictable events. Concerning clinical decision-making, discrimination is arguably the most relevant indicator for model performance. The AUC of the five annual prediction models of the original INFLUENCE nomogram which is exclusively concentrating on the endpoint LRR starts with 0.84 for the first year and decreases to 0.62 in the fifth year [11]. A direct comparison to the AUC values of the INFLUENCE 2.0 model is not possible due to differences in outcome definition (i.e., the original INFLUENCE nomogram predicted the risk of LRR in a given year, assuming the patient was event-free at the start of that year). While discerning high- and low-risk patients for SP seems to be difficult reflected by an AUC between 0.6 and 0.7 for all tested approaches, all other adjusted AUC values reported in this paper were found to be higher than 0.7, indicating a fairly good discriminative ability of the new INFLUENCE 2.0 model. Notwithstanding this, our study shows the importance of finding the optimal statistical approach and model architecture:

In contrast to the logistic regression model of the original INFLUENCE nomogram, a Cox regression-based approach offers the advantage that it can deal with censoring and make time-dependent predictions for periods of variable length in just one model. However, it is based on the proportional hazards assumption and, therefore, cannot incorporate changing event rates over time as easily as the other modeling approaches, which might be the reason that most of the time it showed the lowest discriminative ability. Technically, this problem should be solved by the parametric spline function. However, when concentrating on the time-dependent performance it is evident that both semiparametric models suffer from a lower level of discriminative ability throughout the whole prediction time compared to the more flexible non-parametric RSF approach, which requires more computational power but is not subjected to any preliminary assumptions. Still, the predictor–outcome relation is unknown for the RSF approach, making it difficult to assess the impact of specific characteristics on the estimated risks. The creation of an online calculator improves the transparency of the RSF approach but is explicitly not meant to be used for what-if scenarios. Concerning calibration, the Cox model showed the highest level of agreement between predicted and observed risks, reflected by an average ICI close to 0. To assess the adequacy of calibration, the ICI values should be compared with the observed absolute event rates which were 2.8%, 3.0%, and 6.3% at year 5 for LRR, SP, and DM, respectively; therefore, an ICI below 0.01 was regarded as adequate. In view of this threshold, the RSF approach’s calibration with for example an ICI up to 0.022 for the prediction of DM at year 5 has to be regarded as suboptimal.

Apart from INFLUENCE, several other interesting prediction tools on breast cancer recurrence have been developed. For instance, Corso et al. [31] also came up with a time-dependent prediction model for LRR. At five years after surgery, the cumulative AUC in their validation cohort was 0.77 for patients with breast-conserving surgery and 0.69 with mastectomy. The RSF-based INFLUENCE 2.0 model is characterized by a 5-year AUC of 0.77 regardless of the primary surgical procedure, indicating a tendency toward better discrimination. Giardielo et al. [32] compared three models predicting the risk of contralateral breast cancer (CBC): the Manchester formula, CBCrisk, and PredictCBC in patients with invasive breast cancer (BC). They used data of 132,756 patients (4682 CBC) from 20 international studies with a median follow-up of 8.8 years. The AUCs at five years were: 0.59 (95% Prediction interval (PI): 0.54–0.64) for CBCrisk, 0.61 (95% PI: 0.59–0.63) for the Manchester formula, 0.63 (95% PI: 0.52–0.74) and 0.59 (95% PI: 0.46–0.71) for PredictCBC-1A (for settings where *BRCA1/2* mutation status is available) and PredictCBC-1B (for the general population), respectively. They concluded that the current CBC risk prediction models provide only moderate discrimination, and the Manchester formula was poorly calibrated. Therefore, the RSF-based INFLUENCE 2.0 model on SP with its adjusted AUC of 0.67 represents an important step toward better risk estimations on this endpoint in the general population, even without information on genetics.

### Limitations and strengths in clinical use

Although the bootstrapped model validation showed adequate performance, some limitations of the INFLUENCE 2.0 model should be taken into account. As stated initially, no patients with neoadjuvant treatment or not invasive in situ tumors were included. Second, the set of predictors was obviously limited to the items collected by the NCR. Other potential predictors such as Ki67 were not registered due to comparability issues caused by differing determination methods of different pathology labs [33]. Moreover, information concerning family history, genetic markers or gene signatures such as Mammaprint® or Oncotype were only available for a small number of patients and could therefore not be included in the analyses. Even though pT3 patients were included in the analysis, the majority (98%) of data used to develop the model comprised pT1 and pT2 patients. The use of imputation techniques to deal with missing data could have resulted in the inclusion of more pT3 patients. However, only 42 pT3 patients, which is equivalent to a 0.3% share of all patients (data not shown), were excluded due to missing data. No other subgroup was misrepresented in our dataset, and the sample size was deemed sufficient to perform a complete-case analysis. Future research is required to broaden the applicability of the INFLUENCE 2.0 model and to improve its performance, e.g., by including some of the above-mentioned additional predictors. Further external validation studies and potential model updates should aim to enable model use for patients who received neoadjuvant treatment or to extend the risk prediction period toward 10 years after primary surgery.

Despite these limitations, INFLUENCE 2.0 in its current state can provide substantial added value for patients, health professionals and the health care system as a whole if it is used to tailor follow-up for patients with curatively treated non-metastatic breast cancer. Using individual risk predictions could effectively contribute to decrease the number of potentially unnecessary follow-up visits for patients at a low risk of recurrence. Thus, the overall sensitivity of the breast cancer follow-up program would increase and psychological stress and costs caused by unnecessary examinations in low-risk patients could be avoided [34]. However, the successful implementation of risk-based follow-up requires a truly shared decision process which currently is often not reflected by clinical reality. A review of 42 studies revealed that patients were insufficiently involved in the decision-making process that affected their follow-up, indicating a need for further improvement [35]. With its easy-to-use online interface, the INFLUENCE 2.0 model might be an important step toward more direct patient participation, as recommended by the 2019 guideline on diagnosis, treatment and follow-up for early breast cancer [36] provided by the European society for medical oncology (ESMO): “The interval of [follow-up] visits should be adapted to the risk of relapse and patients’ needs” [7, 36]. Following this recommendation, the risk estimations provided by INFLUENCE 2.0 do not necessarily have to be used together with strict thresholds to discern between high and low-risk patients who should or should not receive follow-up, but can serve as a reliable source of information to find the optimal follow-up strategy, which also has to account for other important factors like the optimal quality of life or patient preference. Further studies are ongoing to assess the impact of implementing the model in the shared decision-making process between clinicians and patients.

## Conclusion

INFLUENCE 2.0 is a flexible risk prediction model for breast cancer recurrence and secondary primary tumors that might be a valuable aid for health care professionals. Together with an appropriate strategy to use its individual, event-specific, time-dependent risk predictions it can support the establishment of a personalized breast cancer follow-up scheme in daily practice.

## Supplementary Information

Below is the link to the electronic supplementary material.

Supplementary file1 (docx 59 KB)

## Data Availability

The data that support the findings of this study were provided by the Netherlands Cancer Registry hosted by the Netherlands Comprehensive Cancer Organisation (IKNL). The data are not publicly available but were used under a specific license for the current study. Data are available from the IKNL upon reasonable request.
