# Supplementary figures and images for: Improved risk estimation of locoregional recurrence, secondary contralateral tumors and distant metastases in early breast cancer: the INFLUENCE 2.0 model
Source: Breast Cancer Res Treat. 2021 Aug 2;189(3):817–26. doi: 10.1007/s10549-021-06335-z (PMC8505302; doi:10.1007/s10549-021-06335-z)

Supplementary data:

Supplementary Figure 1: Flowchart of included patients


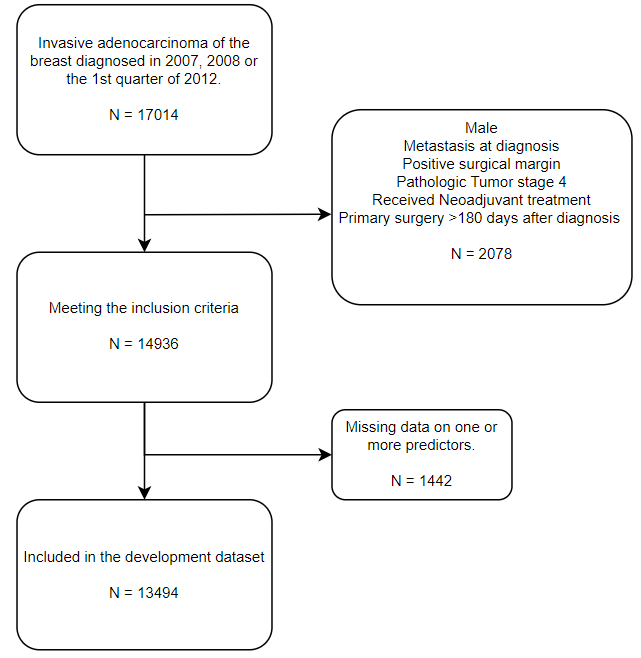

Supplement: Supplementary file 1 — Supplementary file1 (docx 59 KB) [file 10549_2021_6335_MOESM1_ESM.docx]
